# Continuous mHealth Patch Monitoring for the Algorithm-Based Detection of Atrial Fibrillation: Feasibility and Diagnostic Accuracy Study

**DOI:** 10.2196/31230

**Published:** 2022-06-21

**Authors:** Onni E Santala, Jukka A Lipponen, Helena Jäntti, Tuomas T Rissanen, Mika P Tarvainen, Tomi P Laitinen, Tiina M Laitinen, Maaret Castrén, Eemu-Samuli Väliaho, Olli A Rantula, Noora S Naukkarinen, Juha E K Hartikainen, Jari Halonen, Tero J Martikainen

**Affiliations:** 1 School of Medicine Faculty of Health Sciences University of Eastern Finland Kuopio Finland; 2 Doctoral School Faculty of Health Sciences University of Eastern Finland Kuopio Finland; 3 Department of Applied Physics University of Eastern Finland Kuopio Finland; 4 Centre for Prehospital Emergency Care Kuopio University Hospital Kuopio Finland; 5 Heart Center North Karelia Central Hospital Joensuu Finland; 6 Department of Clinical Physiology and Nuclear Medicine Kuopio University Hospital Kuopio Finland; 7 Department of Emergency Medicine and Services Helsinki University Hospital and University of Helsinki Helsinki Finland; 8 Heart Center Kuopio University Hospital Kuopio Finland; 9 Department of Emergency Care Kuopio University Hospital Kuopio Finland

**Keywords:** atrial fibrillation, heart rate variability, HRV, algorithm, stroke, mobile health, mHealth, Awario analysis Service, screening, risk, stroke risk, heart rate, feasibility, reliability, artificial intelligence, mobile patch, wearable, arrhythmia, screening

## Abstract

**Background:**

The detection of atrial fibrillation (AF) is a major clinical challenge as AF is often paroxysmal and asymptomatic. Novel mobile health (mHealth) technologies could provide a cost-effective and reliable solution for AF screening. However, many of these techniques have not been clinically validated.

**Objective:**

The purpose of this study is to evaluate the feasibility and reliability of artificial intelligence (AI) arrhythmia analysis for AF detection with an mHealth patch device designed for personal well-being.

**Methods:**

Patients (N=178) with an AF (n=79, 44%) or sinus rhythm (n=99, 56%) were recruited from the emergency care department. A single-lead, 24-hour, electrocardiogram-based heart rate variability (HRV) measurement was recorded with the mHealth patch device and analyzed with a novel AI arrhythmia analysis software. Simultaneously registered 3-lead electrocardiograms (Holter) served as the gold standard for the final rhythm diagnostics.

**Results:**

Of the HRV data produced by the single-lead mHealth patch, 81.5% (3099/3802 hours) were interpretable, and the subject-based median for interpretable HRV data was 99% (25th percentile=77% and 75th percentile=100%). The AI arrhythmia detection algorithm detected AF correctly in all patients in the AF group and suggested the presence of AF in 5 patients in the control group, resulting in a subject-based AF detection accuracy of 97.2%, a sensitivity of 100%, and a specificity of 94.9%. The time-based AF detection accuracy, sensitivity, and specificity of the AI arrhythmia detection algorithm were 98.7%, 99.6%, and 98.0%, respectively.

**Conclusions:**

The 24-hour HRV monitoring by the mHealth patch device enabled accurate automatic AF detection. Thus, the wearable mHealth patch device with AI arrhythmia analysis is a novel method for AF screening.

**Trial Registration:**

ClinicalTrials.gov NCT03507335; https://clinicaltrials.gov/ct2/show/NCT03507335

## Introduction

### Background

Atrial fibrillation (AF) is globally the most common arrhythmia associated with significant morbidity and mortality, imposing a significant burden on patients, public health, and the health care budget [1,2]. The detection of AF is important in the decision to initiate anticoagulation therapy to prevent thromboembolic events [3,4]. Nonetheless, AF detection is still a major clinical challenge as AF is often paroxysmal and asymptomatic [5-8]. AF screening recommendations include opportunistic or systematic screening in patients ≥65 years of age or in those individuals with other characteristics pointing to an increased risk of stroke [3]. The European Heart Rhythm Association recommends multiple time points or over a prolonged time to increase diagnostic yield in AF screening with digital devices [9]. The popularities of well-being and taking personal responsibility for one’s own health are reflected in the continuous development and growth of mobile health (mHealth) technologies. There are currently more than 400 wearable activity monitors and more than 100,000 mHealth apps already available [10]. mHealth technologies could provide an additional opportunity to diagnose AF, particularly its paroxysmal and asymptomatic forms [11]. Most mHealth technologies designed for AF detection include some form of automatic AF detection algorithm [12]. These novel mHealth technologies could provide a cost-effective solution for AF-screening [13].

### Objective

A highly popular and evolving mHealth technology could reach the population to be screened at a relatively low cost and with little logistical effort, since well-being and personal health care monitoring is already a part of everyday life in many individuals. Several of these new devices produce heart rate variability (HRV) data, which have been widely used to assess a variety of well-being measures such as recovery and stress monitoring [14]. Furthermore, HRV monitoring could also be suitable for other health-related measurements such as AF detection [10,15]. Algorithm-based rhythm monitoring with devices designed for well-being and health-related measurements could enable straightforward, cost-effective, and reliable methods for AF screening. The specific aims of this study were to (1) evaluate the feasibility and quality of the HRV data using a single-lead electrocardiogram (ECG)–based mHealth patch device, and (2) assess the accuracy of an artificial intelligence (AI) arrhythmia detection algorithm in AF screening with 24-hour monitoring.

## Methods

### Study Setting and Participant Recruitment

The study was conducted as a single-center study between April 2018 and December 2019 in Kuopio University Hospital. The study patients were recruited from the hospital emergency care department. The inclusion criteria were AF or sinus rhythm (SR) based on a 12-lead resting ECG recorded during admission to the hospital. The exclusion criteria were as follows: (1) estimated stay in the hospital <24 hours; (2) BMI ≥35kg/m2; (3) left bundle branch block or right bundle branch block; (4) implanted cardiac pacemaker; and (5) a medical condition requiring immediate treatment. The clinical characteristics and symptoms prior to hospital admission of the patients were collected using a standardized data collection protocol and confirmed or complemented from the medical records. All participants provided written informed consent to participate in the study. All data were pseudonymized. Each subject was given an ID number, and the data were kept locked and encrypted in the study files. The measurement data did not include personal data. The research data collected in the study will be treated confidentially as required by the Personal Data Act.

### HRV Measurements and Analysis

**Figure 1:**
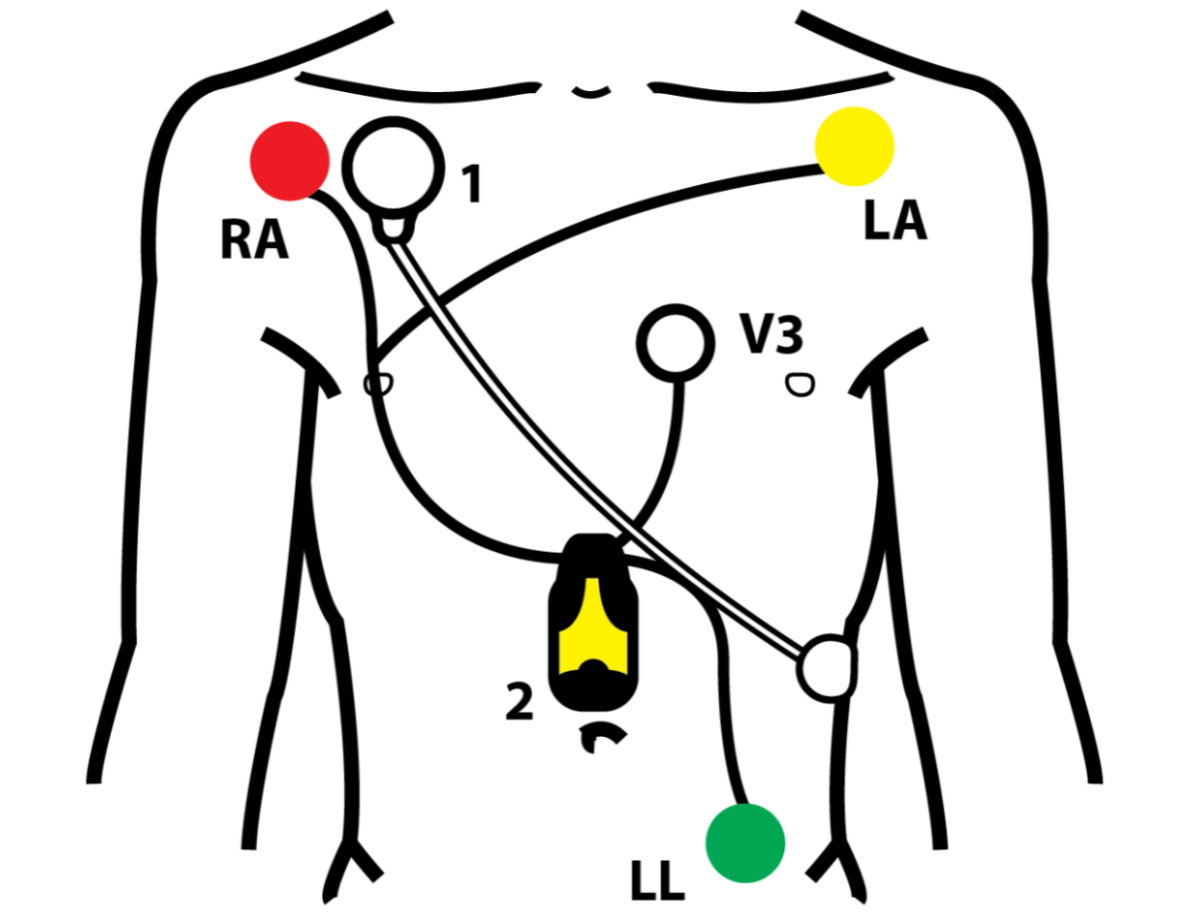
Heart rate variability (HRV) and electrocardiogram (ECG) recordings. Single-lead ECG-based HRV recording (1) and 3-lead Holter ECG recording (2). LA: left arm; LL: left limb; RA: right arm; V3: V3 lead in 12-lead ECG.

A single-lead ECG-based HRV device (Firstbeat Bodyguard 2, Firstbeat Technologies) was applied to the patient’s chest with 2 adhesive patches as shown in Figure 1. The Firstbeat Bodyguard 2 device records ECG data, from which it stores beat-to-beat R-R intervals (time elapsed between two successive R-waves) to allow an HRV assessment. The target time for HRV measurement was 24 hours. Simultaneously registered 3-lead Holter ECG recording (Faros 360, Bittium) was used as the “gold standard” for rhythm classification (Figure 1). Both devices stored the data in the internal memory of the device.

A commercial arrhythmia analysis software (Awario, Heart2Save) was used for automatic AF screening from the HRV data. The AI arrhythmia detection algorithm classified the HRV data in 30-second time windows into 3 categories: SR, AF, and uninterpretable. The accuracy of the AI-based rhythm classification from the HRV recording was further assessed by comparing it with the gold standard Holter ECG recording. Holter ECG recordings were analyzed using a Medilog Darwin Professional V2.8.1 software (Schiller Global). ECG recordings were reviewed independently by 4 investigators blinded to the initial 12-lead ECG and classified into either AF or non-AF rhythms.

### Statistical Analysis

The size of the study sample was estimated as 200 observations with an assumed sensitivity of 95% and with a 3% margin error. The AF and control groups were compared using *t* test for continuous variables and χ2 test or Fisher exact tests for dichotomous variables. All HRV data were analyzed by the AI arrhythmia detection algorithm in 30-second time windows. The performance of the AI arrhythmia detection algorithm in AF detection from HRV recordings was quantified using accuracy, sensitivity, specificity, negative predictive value (NPV), and positive predictive value (PPV). The performance of the AI arrhythmia detection algorithm was tested by (1) detecting AF per patient (subject-based) and (2) total accumulated AF duration across all patients (time-based). All significance tests were 2-tailed, and *P*≤.05 was considered statistically significant. The data were analyzed using IBM SPSS statistics software, version 27. This study was registered in the ClinicalTrials.gov database (NCT03507335).

### Ethics Approval

The study design was approved by Research Ethics Committee of the Northern Savo Hospital District (347/2018).

## Results

### Study Population

**Figure 2:**
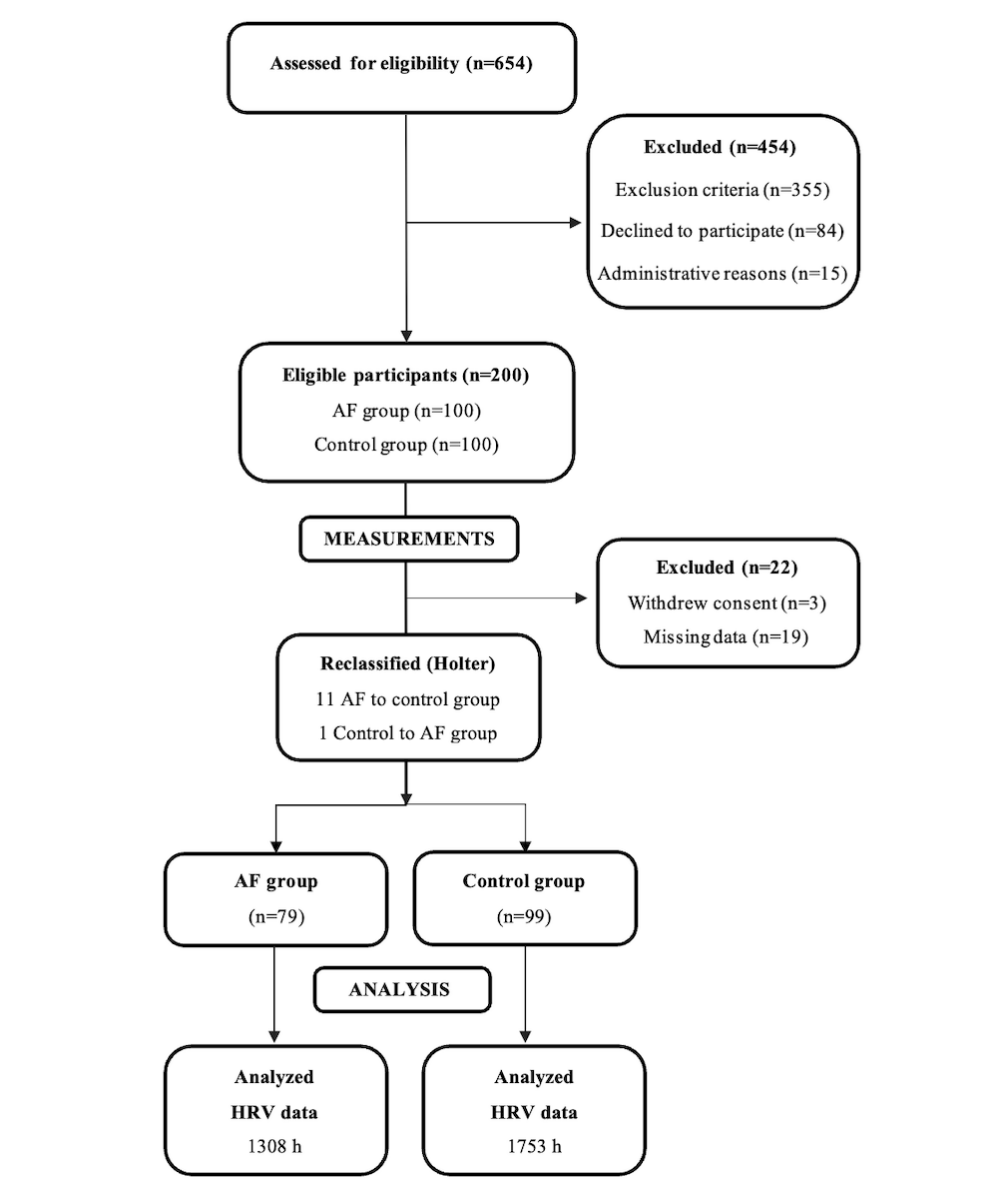
Study flow chart. AF: atrial fibrillation; HRV: heart rate variability.

A total of 654 patients were assessed for eligibility. In the initial assessment for the study patients, 454 (69.4%) patients were excluded for the reasons summarized in Figure 2. A total of 200 eligible patients were included in study, of which 100 (50%) were assigned to the AF group and 100 (50%) with SR to the control group. Of the 200 eligible participants, 22 (11%) were further excluded (n=19, 10% were excluded due to missing data and n=3, 1.5% withdrew their consent). In addition, the rhythm of some patients had converted from the time of 12-lead ECG recording prior to study measurements. Consequently, the final rhythm classification made from Holter ECG recording reclassified 11 patients from the AF group into the control group and 1 patient from the control group into the AF group. Thus, the final study population consisted of 178 patients, of whom 79 (44%) were in the AF group and 99 (56%) in the control group.

Compared to the control group, patients with AF were older (77, SD 10 vs 68, SD 15 years; *P*<.001), presented more often with a history of paroxysmal AF (*P*<.001), hypertension (*P=.*02), congestive heart failure (*P<.*001), and previous heart surgery (*P=.*02), and were more often receiving anticoagulation (*P<.*001), beta-blocker (*P<.*001), and digoxin (*P=.*005) therapy. Patients with AF also more often reported palpitations (*P=.*005) and respiratory distress (*P<.*001), compared to the control group. These groups did not differ statistically with respect to the other recorded parameters (Table 1).

**Table 1:** Patient demographics.

Characteristics and demographics			Control group (n=99)		AF^a^ group (n=79)		Significance (2-sided)	
Age (years), mean (SD)			68 (15)		77 (10)		<.001	
BMI (kg/m^2^), mean (SD)			26 (4)		27 (4)		.16	
**Sex, n (%)**								.09
	Male, n (%)	40 (40)		42 (53)				
	Female	59 (60)		37 (47)				
**Medical history, n (%)**								
	Earlier AF episode	21 (21)		63 (80)		<.001		
	Coronary heart disease	27 (27)		26 (33)		.41		
	Diabetes mellitus	21 (21)		19 (24)		.65		
	Hypertension	57 (58)		59 (75)		.02		
	Congestive heart failure	12 (12)		39 (49)		<.001		
	Previous heart surgery	7 (7)		15 (19)		.02		
**Medication, n (%)**								
	Anticoagulation therapy	26 (26)		67 (84)		<.001		
	Beta blocker	42 (42)		56 (71)		<.001		
	Digoxin	4 (4)		13 (17)		.005		
	Antiarrhythmic medication	1 (1)		1 (1)		>.99		
**Symptoms prior to hospital admission (%)**								
	Decrease in general condition	56 (57)		45 (57)		.96		
	Fatigue	51 (52)		47 (60)		.29		
	Palpitations	23 (23)		34 (43)		.005		
	Respiratory distress	26 (26)		39 (49)		<.001		
	Chest pain	17 (17)		18 (23)		.35		

^a^AF: atrial fibrillation.

### Quality of the HRV Data

**Figure 3:**
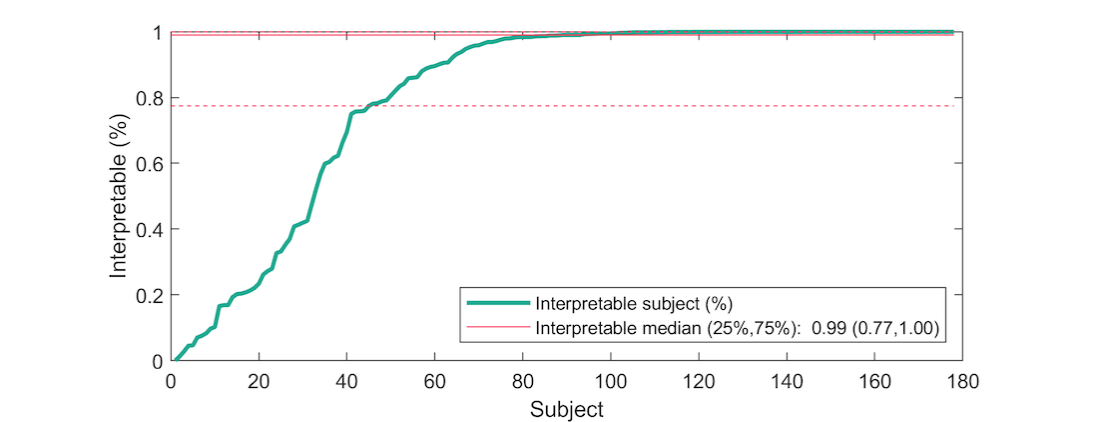
Percentage of interpretable heart rate variability data in individual subject recordings. Subjects sorted by using automatic quality values.

In the automatic analysis of HRV data (all subjects), 3099 hours out of 3802 hours (81.5%) were estimated as being interpretable. In Holter ECG recordings, 3723 hours were deemed interpretable. Based on the evaluation of the AI arrhythmia detection algorithm, 1308/1543 hours (84.8%) of the AF group recordings and 1753/2180 hours (80.4%) of the control group recordings were estimated as being interpretable. The subject-based median for interpretable data was 99% (25th percentile=77% and 75th percentile=100%) (Figure 3).

### Accuracy of the AI Arrhythmia Detection Algorithm When Interpreting HRV Data

The accuracy of rhythm analyses was evaluated from the data deemed interpretable in both HRV and ECG recordings (3062 hours). The AI arrhythmia algorithm detected AF correctly in all the patients in the AF group and suggested the presence of AF in 5 patients in the control group (false-positive AF detection), resulting in a subject-based AF detection accuracy of 97.2% (173/178), a sensitivity of 100% (79/79), a specificity of 94.9% (94/99), PPV of 94.0% (79/84), and NPV of 100% (94/94) (Table 2). The values of the time-based accuracy, sensitivity, and specificity of the AI arrhythmia detection algorithm for identifying AF were 98.7% (3022/3062 hours), 99.6% (1304/1308 hours) and 98.0% (1718/1753 hours), respectively; the PPV was 97.4% (1304/1339 hours) for detecting the presence of AF, and the NPV was 99.7% (1718/1723 hours) for its absence (Table 3). We inspected for the reasons for false-positive AF detections, which were as follows: frequent (>10,000 per 24 hours) ventricular extrasystoles, atrioventricular block, a short section (12 minutes) with many ventricular extrasystoles, and in 2 cases they were attributable to undetected QRS complexes (Q wave, R wave, and S wave).

**Table 2:** Subject- and time-based AF detection accuracy, sensitivity, and specificity based on artificial intelligence arrhythmia detection algorithm.

Algorithm types	Accuracy, n/N (%)	Sensitivity, n/N (%)	Specificity, n/N (%)
Subject-based algorithm	173/178 (97.2)	79/79 (100)	94/99 (94.9)
Time-based algorithm (h)	3022/3062 (98.7)	1304/1308 (99.6)	1718/1753 (98.0)

**Table 3:** PPV^a^ and NPV^b^ based on artificial intelligence arrhythmia detection algorithm.

Algorithm types	PPV, n/N (%)	NPV, n/N (%)
Subject-based algorithm	79/84 (94.0)	94/94 (100)
Time-based algorithm (h)	1304/1339 (97.4)	1718/1723 (99.7)

^a^PPV: positive predictive value.

^b^NPV: negative predictive value.

## Discussion

### Principal Findings

We demonstrated the good feasibility and diagnostic performance of the mHealth patch device and AI arrhythmia detection algorithm for AF detection. The main findings were as follows: (1) the mHealth patch device designed for well-being and personal health care measurements produced interpretable HRV data from 24-hour recording; and (2) the AI arrhythmia detection algorithm achieved an accurate AF detection from the HRV data.

### Comparison to Prior Work

ECG-based wearables, especially adhesive patches, are probably the most valuable approach for tracking HRV data [16]. In previous studies using adhesive patch devices, 92%-99% of the ECG or ECG-based HRV data have been reported to be analyzable with recording times ranging from 12 hours to several days [17-19]. In line with these reports, in our study of the HRV data produced by the ECG-based mHealth patch, 82% were analyzable, and in the subject-based analysis, the median of the data to be analyzed was 99%. However, interindividual differences were observed in the HRV data quality. In 32/178 patients (18%), less than 50% of the data were interpretable (Figure 3). According to a recent systematic review, although the correlation between HRV as measured by Holter and ECG-based wearable devices was excellent at rest, it declined down to 0.85 during movement or exercise [20]. In our study, mobilization of the study patients was not restricted. Despite this, the mHealth device provided high-quality ECG data for HRV monitoring.

The diagnostic accuracy of the AI arrhythmia detection algorithm used in this study to detect AF was comparable to other screening methods and devices. In the previous studies, the sensitivity of automatic AF detection ranged from 67% to 100% and the specificity from 84% to 100% depending on the mobile or digital technology and strategy used [21-36]. In our study, the sensitivity and specificity of AF detection were 100% and 94.9%, respectively. Although the new mHealth screening methods have a high sensitivity and specificity for AF detection, fals-positive AF detections (false alarm) remain a major concern, especially when low-risk individuals use these devices [37]. A false alarm can lead to anxiety, additional health care costs, and possibly even inappropriate treatment [37]. Nonetheless, it should be noted that some false alarms may be due to another arrhythmia or some other pathological condition, and in these cases, screened individuals may benefit from the AF screening program [38]. Our results support this idea since 3 (60%) of the 5 “false alarms” in our study were due to a true arrhythmia. Further, the threshold for confirming the arrhythmia diagnosis by a health care professional from an ECG recording should be low.

### Future Directions

The current guidelines recommend AF screening for individuals at an elevated risk of stroke, such as those over 65 years of age [3]. However, there is no unambiguous guidance on the actual screening strategy to be adopted. According to the current guidelines of the European Society of Cardiology, the development of wearable technology is likely to provide cost-effective and practical alternatives for the detection of AF [3]. The popularity of well-being and personal health care monitoring is reflected in the continuous development and growth of the mHealth technologies [10]. However, most of these techniques have not been validated for clinical purposes [10]. Therefore, before using mHealth technologies, one should carefully consider whether the technology in question has been validated for clinical use [14]. Novel methods, such as the AI arrhythmia analysis used here, could provide reliable AF screening when combined with technologies designed for well-being and personal health care measurements. Firstly, a longer duration of rhythm monitoring is more sensitive compared to single recording in AF screening [39]. Secondly, the advantage of automatic AF screening helps to identify arrhythmia episodes from long-term ECG or HRV data, particularly in patients with paroxysmal and asymptomatic AF. Consequently, these techniques could enable the screening of patients at high risk for AF and stroke and the allow follow-up of patients after cardioversion and ablation as well as those recovering from stroke or transient ischemic attack.

### Study Limitations

We acknowledge some limitations in our study. First, the presence of morbid obesity could degrade the signal quality and thus produce more failed measurements. In addition, in the right bundle branch block and left bundle branch block cases, the presence of broad 2-peak QRS complexes could result in a false R-R interval, and thereby, an erroneous AF detection by the automatic algorithm. For these reasons, these patients were excluded, and further studies will be needed in these subgroups. In the future, algorithm-based detection of AF with HRV mHealth patch devices will need to be conducted in an out-of-hospital setting to assess the signal quality and accuracy of AF detection in these scenarios.

### Conclusions

The mHealth patch device provided good quality HRV data for accurate automatic AF detection from 24-hour monitoring. The AI arrhythmia detection algorithm detected AF with high accuracy, sensitivity, and specificity. Thus, the wearable mHealth patch device with AI arrhythmia analysis could represent a new, promising method for AF screening.

## Abbreviations

AF: atrial fibrillation
AI: artificial intelligence
ECG: electrocardiogram
HRV: heart rate variability
mHealth: mobile health
NPV: negative predictive value
PPV: positive predictive value
SR: sinus rhythm
